# Attending a one-to-one child-centered movement therapy program improves multiple outcomes among children with neurodevelopmental disabilities: an exploratory prospective cohort study

**DOI:** 10.3389/fped.2025.1623686

**Published:** 2025-11-13

**Authors:** Mojgan Gitimoghaddam, William H. McKellin, Lise Olsen, Anton R. Miller, Vivien Symington, Jean-Paul Collet

**Affiliations:** 1 Department of Pediatrics, University of British Columbia, Vancouver, BC, Canada; 2 BC Children’s Hospital Research Institute, Vancouver, BC, Canada; 3 Department of Anthropology, University of British Columbia, Vancouver, BC, Canada; 4 School of Nursing, University of British Columbia, Okanagan, BC, Canada; 5 Club Aviva Recreation Ltd., Coquitlam, BC, Canada

**Keywords:** physical activity, neurodevelopmental disability, children, child development, motor skill, autism, personalized approach, scaffolding

## Abstract

**Introduction:**

Children with neurodevelopmental and/or intellectual disabilities/disorders (NDID) often face barriers that limit their participation in typical school and community activities alongside their peers. Adapted, community-based physical activity programs may help bridge this gap by offering safe and engaging environments that promote integration, socialization, and learning. This study aimed to evaluate the potential effects of the Empowering Steps Movement Therapy (ESMT™) one-to-one personalized physical activity program on children and youth with NDID.

**Method:**

A prospective cohort study was conducted to collect information regarding the changes in participants’ motor skills (measured by BOT-2-SF), social and leisure adaptive skills (measured by ABAS-II), community and home participation (measured by PEM-CY), and quality of life (measured by KIDSCREEN-27). Forty-two children and youth with NDID attending the ESMT™ program were followed for 12 months with follow-up every 3 months. A linear mixed-effects (LME) model was used to analyze longitudinal changes, as well as studying the influence of children's and families' baseline characteristics on the outcomes.

**Results:**

For motor skills, children showed a small but consistent positive trend in standardized motor skills (BOT-2-SF) over time, averaging 0.17 points per month (95% CI: −0.01 to 0.34; *p* = 0.06), with a slightly higher gain among autistic children (0.20 points; 95% CI: −0.03 to 0.43; *p* = 0.08). Positive trends were also observed in home and community participation. However, improvements were minimal for social adaptive skills, and a slight decline was noted in leisure adaptive skills and quality of life. Several potential confounders including parental education, socioeconomic status, and participation in other community-based programs were examined, indicating that some may play a significant role. Notably, the type of underlying condition contributing to neurodevelopmental disability appeared to be an important effect modifier for both motor skills and social adaptive skills.

**Conclusion:**

Despite the small sample size, this study suggests that individualized, one-to-one physical activity programs which provide individualized scaffolding tailored to each child's unique needs, may yield meaningful improvements across multiple domains in children with NDID. Larger studies are warranted to validate these findings and further support the adoption of personalized, socially engaging approaches in therapeutic physical activity settings.

## 1 Introduction

Neurodevelopmental and/or intellectual disabilities/disorders (NDID) consist of a range of diagnoses and functional impairments of a neurological origin that can be experienced as functional delays in developmental milestones such as language, communication, social skills, intellect, executive functioning, and motor development (1–4). Examples of NDIDs include autism spectrum disorders (ASD), attention deficit/hyperactivity disorder (ADHD), Down syndrome, and fetal alcohol spectrum disorder (FASD). The prevalence of NDID across developed countries in children and youth 18 years of age and younger ranges from 8% to 15% (5–7).

Because of their limitations, children with NDID are often excluded from participating with their peers in typical school and community activities. This may lead to isolation and loneliness which can result in limiting their learning abilities and development potential, with long term consequences for their physical and mental health (8). Adapted community-based physical activity programs may help to address the challenge of isolation by providing a safe and playful environment to facilitate integration, socialization and learning.

The literature on the effects of physical activity programs (PAP) on children and youth with NDID has shown the positive impacts of physical activity on a variety of outcomes such as physical literacy (9), social integration (10, 11), friendship development (12–14) and cognitive functioning (15–17). However, the majority of these studies are limited due to the high likelihood of the Hawthorne effect as well as observational biases in context of controlled trials and quasi-experimental studies. Other limitations include short follow-up periods and a limited range of outcomes. For example, in the studies conducted by Ketcheson et al. 2021 (18) and Zhao & Chen 2018 (19), follow-up measures were taken one-week after the intervention. Furthermore, studies such as Sotoodeh et al. 2017, Zachor et el. 2017 and Caputo et al. 2018 (20–22) did not have any follow-up measures, which raises concerns regarding the results reported. With respect to limited outcomes, many studies measured only one outcome, potentially overlooking other outcomes that could have been impacted. For instance, many studies collected data on just one type of outcome measures among motor development, social skills, quality of life (QoL), community activities, leisure or home participation. Others only collected specific measures of motor progress (23), or did not consider how social communication may have been impacted (24).

In 2015, we published a retrospective study of a gymnastic-based movement therapy program for children with NDID called Empowering Steps Movement Therapy (ESMT™) (25). Following this study, we conducted an exploratory prospective cohort study that is described in this current paper using standardized measurement tools to confirm previous findings and develop more complex analyses that will be reported in this paper. We also attempted to avoid the weaknesses of previous studies by studying children in a more natural environment (preventing Hawthorn effect), without observational biases and minimizing selection biases.

The aim of this study was to investigate the possible effects of the ESMT™ program on children and youth with NDID. The effects of the program were assessed for numerous outcomes, including their impact on children's motor skills, social adaptive skills, leisure adaptive skills, community participation, and their QoL. When changes were observed, we sought to determine the reasons, identify the main factors involved, and understand the dynamics of the change process.

The study was reviewed and approved by the University of British Columbia/Children's and Women's Health Center of British Columbia Research Ethics Board (Certificate numbers: H14-02258).

## 2 Materials and methods

### 2.1 Design

To study the impact of attending the ESMT™ program, we conducted a prospective cohort study from June 2015 to August 2018 with children with NDID, attending the ESMT™ program. Both children who were attending the program when the study was initiated, as well as those who joined the program after study initiation were invited to participate for a 12-month follow-up study that included data collection every 3 months. Children and youth with different durations of previous PAP exposure were included to provide information regarding the trends of improvement in children with different levels of exposure to the program.

Enrollment into the ESMT™ program followed a standard procedure, including an intake call, completion of an intake form, review by the ESMT™ team, an initial assessment conducted by both a supervisor and a therapist to determine baseline motor and communication needs, and assignment to a regular therapist with a consistent schedule.

After parents signed the consent to participate, every participant was assessed at baseline (when they joined the study) and every 3 months (within the time window of ± 2 weeks) using a standard motor scale and standard questionnaires to assess psychosocial outcomes, functional abilities and QoL. This process was the same for prevalent and incident cases.

### 2.2 Participants

42 children and youth with NDID who attended the ESMT™ program participated in the study. Children were eligible to participate if they were 5–15 years of age at the time of recruitment, had any type of NDID and a minimum level of motor function to perform the Bruininks-Oseretsky Test of Motor Proficiency-Short form (BOT-2 SF); children without a formal clinical diagnosis were also included if they had clinically observed delays and met the other inclusion criteria. They were excluded if they were non-ambulatory or diagnosed with neurodegenerative disorders because motor functions progressively deteriorate in these children (thus limiting our ability to study a possible improvement in context of the ESMT™ program), and/or if their parents/guardians were not able to provide consent or speak/understand English. No specific inclusion or exclusion criteria were applied based on cognitive ability. Given that comprehension and attention may influence motor test performance, adapted administration procedures have been utilized (e.g., demonstrations, simplified instructions, and visual modeling) to support understanding and participation. As this was a single site real-world study, we applied minimal selection criteria to include the maximum number of children enrolled in the ESMT™ program without age or diagnosis restrictions. We examined age as a potential effect modifier and confounder; we also studied the effects among children with ASD.

### 2.3 Study procedures

The index date for each child was the time parents signed the consent form for study participation. At baseline, parents completed the demographic questionnaire and three standardized scales (Adaptive Behaviour Assessment System-Second edition (ABAS-II), Participation-Environment Measure for children and Youth (PEM-CY), KIDSCREEN-27 to assess the child's functionality in different domains. We also assessed the motor function of the participants using the BOT-2 SF at the program site or at other locations if this was parents' preference. Finally, we measured the baseline severity score of children with ASD by Social Communication Questionnaire (SCQ)—Current version. The SCQ has cross-cultural validity and diagnostic validity (26). This tool also has a high internal consistency, with Cronbach's alpha of 0.89 for non-verbal children and 0.94 for verbal children (27).

Follow-up assessments were conducted every 3 months, including the BOT-2-SF, ABAS-II, PEM-CY and KIDSCREEN-27 but not including the demographics and SCQ. The 3-month assessments were conducted four times.

Motor skills assessments were conducted by the research staff independently from the program staff and therapists.

Parents could complete the self-report forms either electronically or paper-based. For electronic self-report, study data were collected and managed using REDCap electronic data capture tools hosted at The BC Children's Hospital Research Institute (28, 29). REDCap (Research Electronic Data Capture) is a secure, web-based software platform designed to support data capture for research studies, providing (1) an intuitive interface for validated data capture; (2) audit trails for tracking data manipulation and export procedures; (3) automated export procedures for seamless data downloads to common statistical packages; and (4) procedures for data integration and interoperability with external sources. Electronic submission enabled parents to complete the questionnaires at their convenience. For those using paper-based reporting, parents completed these questionnaires while their child was at the training session or took them home and brought them back at a subsequent session. The parents had the opportunity to contact the researcher to ask any questions they might have in completing the questionnaires.

### 2.4 Intervention and setting (ESMT™ program)

The current study was conducted at Club Aviva Recreation Ltd. (Club Aviva), which is a gymnastics facility for all abilities located in the Greater Vancouver Regional District. The ESMT™ program, founded in 2002, was a structured one-to-one movement therapy that served a broad range of children with different types of NDIDs. The program occurred in a fully inclusive gymnastics environment alongside recreational and competitive gymnastics programs. The three main components of the ESMT™ process include motor development, emotional engagement, and social attachment. Motor activities are utilized as the medium to impact the child's emotional state and their ability to develop social attachment through building relationships with other children and coaches. The ESMT™ program is highly structured by the ESMT™ curriculum and assessment scale (see Supplementary Material—Description of the ESMT™ Process). Each stage includes 20 scaffolded motor skills. The Motor scale itself includes 10 stages of motor development and promotes each child's development by setting individualized goals. Any child starting the program can be assessed and assigned to a stage that corresponds to their current level of motor functioning. Further assessments are conducted semi-annually, to update the child's plan appropriately (Supplementary Material—Case Study).

The ESMT™ program follows a proprietary motor scale designed to guide implementation in a consistent yet personalized manner. Children are re-evaluated biannually, and outstanding skills are prioritized for subsequent sessions until the next biannual assessment. Because each level on the scale is carefully defined, the program can be precisely adapted to each child's abilities and specific needs, providing structured scaffolding within the child's zone of proximal development. Consequently, children cannot progress to the next stage until all motor skills from their current stage are completed. This process ensures both consistency in practice and individualization in progression.

The training sessions were usually 30, 45, or 60 min in duration and typically took place one to three times per week depending on the needs and circumstances of each family. Most participants attended one 60-minute session per week between September and June, usually with the same therapist and at the same time slot throughout this period. Some families opted for two sessions per week, although the cost of one-to-one therapy (approximately $100 per hour per child) was often a limiting factor. Most ESMT™ staff members held BSc degrees in programs such as Kinesiology or Health Science. In order to be certified as an ESMT™ therapist, staff were required to receive standard training by an experienced program supervisor for a minimum of 480 h, including practical and theory evaluations.

Fidelity to the ESMT™ curriculum protocol was strictly implemented. Each therapist was trained on the ESMT™ Motor Scale during the 480-hour certification period and was required to complete 24 theory modules and pass a quiz for each module. Trainees were formally evaluated on their motor program delivery halfway through and again at the end of training. The ESMT™ training emphasizes coaching excellence and adherence to the curriculum protocol to ensure consistent practice among therapists. Supervisors are available to provide guidance and observe sessions when concerns arise or when new staff require support.

At the same time, the ESMT™ approach is highly individualized at the child level. Each child's program is tailored to their specific skills, interests, and goals within the structure of the standardized curriculum. Therefore, while fidelity is closely monitored at the program and coach levels, no inter-rater reliability checks are performed for individualized child-level adjustments.

While the primary goal of the ESMT™ was to improve the motor function of children, ESMT™ therapists also incorporated communication, social, and behaviour support. This method follows Vygotsky's theory, where learning happens with help from a more skilled person. Trainers supported children step by step in their ZPD to improve skills through social interaction. The main independent variable in the current study was the number of months that a child was exposed to the ESMT™ program.

### 2.5 Outcomes assessments

#### 2.5.1 Primary outcomes

- Primary outcomes included: Motor skills using *BOT-2 SF*; Social adaptive skills and Leisure adaptive skills which were both assessed by ABAS-II.

BOT-2 (30–32) is a standardized and norm-referenced tool for psychomotor assessment of individuals ranging from ages 4 – 21 (30). It was chosen as primary outcome because it is the most likely outcome to improve in context of a physical activity program. It is also the most frequent outcome chosen in other studies assessing the effects of PAPs. BOT-2 contains four domains: Fine manual control, Manual coordination, Body coordination, and Strength and Agility. One of the two forms of BOT-2 is the BOT-2 short form (BOT-2-SF). 14 test items are selected proportionally from the subsets of the Complete Form to compose the Short Form (31). Three types of reliability are incorporated into the BOT-2-SF: inter-rater reliability, test-retest reliability, internal consistency reliability. Inter-rater reliability is above 0.90 for the BOT-2-SF. Test-retest reliability is also excellent for the BOT-2-SF (mid to upper 0.80s). Internal consistency estimates are high for the Short Form as well, with correlations falling within the range 0.60 to 0.92 (32). In addition, content validity is appreciable for this tool as well.

ABAS-II (33–36) measures adaptive behaviour and activity of daily living. It is a norm-referenced, comprehensive tool used to assess adaptive skills and behaviour in three domains: Conceptual, Social and Practical. The assessment is applicable across the population, with ages ranging from birth through 89. We used the parent report form for 5–21 years of age. ABAS-II should take approximately 20–30 min to complete. ABAS-II has good validity and excellent reliability. In this study, we report the findings for Social and Leisure adaptive skills that are two skill areas under the social domain. Reliability scores for the social adaptive domain fall between 0.91 and 0.98, with the 10 individual skills ranging from 0.80 to 0.97. Social adaptive skills and leisure adaptive skills were also chosen as primary outcomes because of their importance in child development (especially according to Vygotsky's sociocultural learning theory), and because the probability of improvement when motor skills improve was perceived high in context of ESMT™ program.

#### 2.5.2 Secondary outcomes

Secondary outcomes included changes in participation in daily activities at home and in the community measured by PEM-CY; and QoL measured by KIDSCREEN-27.

PEM-CY (37, 38) is a parent-reporting tool. It evaluates participation across home, school, and community settings, accounting for the environmental factors specific to each context. PEM-CY is appropriate for use in children and youth ranging from 5 to 17 years old, including those with or without disabilities. PEM-CY shows good to strong correlation with other standard scales assessing environment (Validity); test-retest in a four-week interval is good [Intraclass Correlation Coefficient (ICC): 0.58–0.95]. Also, there is significantly notable effect of disability across all settings, which is consistent within age-intervals and consistent with other literature with similar reports. For this study, we report on the participation at home and in the community. Internal consistency coefficients for “Participation frequency” at home is evaluated to be 0.59. Test-retest reliability is good at 0.84. Internal consistency coefficients for “Participation frequency” in the community is evaluated to be 0.70 (38). Test-retest reliability is fairly good at 0.79.

KIDSCREEN-27 (39–42) was developed as collaborative projects in Europe. The KIDSCREEN instrument is designed to be used for children and adolescents in the age range of 8–18 years old.

KIDSCREEN-27 takes about 10–15 min to complete, developed as a slightly shorter version of the KIDSCREEN-52. KIDSCREEN-27 encompasses 5 dimensions with profile information for Psychological Well-being, Physical Well-being, Autonomy & Parent Relations, School Environment, and Social Support & Peers. The KIDSCREEN instrument has satisfactory to excellent properties such as internal consistency, test-retest reliability, along with validity. Cronbach's alpha for the internal consistency falls in the range 0.80–0.84 for KIDSCREEN-27. Test-retest reliability has ICCs in the range of 0.61–0.74 (39).

### 2.6 Data analysis

Because this analysis was mostly exploratory, we focused more on trends and confidence intervals, rather than strict *p*-values. The small sample size may have limited the statistical power to detect modest effects, and thus borderline *p*-values should be interpreted with caution. Also, because of the small sample size, we did not adjust for multiple comparisons and considered alpha = 0.1 as *p*-values threshold of interest for the discussion. This approach was chosen to be more inclusive than exclusive, to avoid prematurely rejecting potentially meaningful associations that may warrant further investigation. As such, none of the conclusions are definitive based on *p*-values but rather point to possible trends that could inform future, larger studies. The analysis was completed using R software.

The study's main independent variable was the number of months that a child was exposed to the program. Covariates consisted of 13 variables that could be confounders based on the knowledge we have of motor skills or social abilities development. Seven continuous variables: age, baseline severity, attendance in other non-PAP group activities (Group-NPAP), attendance in other group-PAP (Group-PAP), attendance in other one-to-one non-PAP (one-to-one-NPAP), attendance in other one-to-one PAP (one-to-one-PAP), and duration of previous attendance in the studied PAP before first assessment, as well as six categorical variables, including gender, diagnosis, family income, father's education, mother's education, and parents' marital status.

In order to deal with the outliers, a sensitivity analysis was conducted by investigating the effects of the outliers and comparing the results of analysis before and after removing them. Also, non-parametric tests to compare variances and IQR were used because they are less sensitive to the presence of outliers.

### 2.7 Drop out and loss to follow-up

There were two types of missing data. Those related to the baseline characteristics possibly due to parents' reluctance with disclosing sensitive information. In the effort of retaining these missing data in the covariates and to examine if there were significant differences between the missing and non-missing reports, a new category for “missing information” was made in the analyses. The second type of missing data was related to the QoL outcome measures. Simple imputations were made by taking the averages of the score from other children. We then conducted a sensitivity analysis to compare the results before and after the imputation.

We obtained summary statistics for the variables in the study to explore distributions and dispersion of the data. Descriptive statistics were also generated on the outcome scores for each assessment time point and the percentage changes between baseline and follow up assessments was computed.

Prior to the main analyses, exploratory tests were conducted between baseline and follow-up assessments to better understand the magnitude of changes for the outcome variables using the Paired t-tests. In the case of violating normality assumption, non-parametric Wilcoxon Signed Rank tests were used after verification of the assumptions.

Using the linear mixed effects (LME) model, we estimated the effect of attending the ESMT™ program on each study outcome, adjusting for possible confounding variables. We also introduced an interaction term in the LME model to assess the possible modification of effect according to different levels of the covariate variables.

## 3 Results

Baseline characteristics of children and their families have been summarized in Table 1.

**Table 1:** Baseline characteristics of study participants**.**

Variables	Count (%)
Gender	
- Male	8 (19%)
- Female	34 (81%)
Diagnosis^a^	
- ASD	26 (62%)
- Down syndrome	1 (2%)
- Other	14 (34%)
- Missing	1 (2%)
Income^a^	
- < $50k	5 (12%)
- $50k - $90k	10 (24%)
- > $90k	12 (29%)
- Prefer not to answer	9 (21%)
- Missing	6 (14%)
Father's education^a^	
- High/Trade	14 (33%)
- University	24 (57%)
- Missing	4 (10%)
Mother's education^a^	
- High/Trade (completed high school or trade school)	12 (29%)
- University (All degrees)	28 (67%)
- Missing	2 (6%)
Marital status^a^	
- Married (Official)	34 (81%)
- Single	6 (14%)
- Missing	2 (5%)
Variables	Mean (SD)
Age (month)	107.12 (35.72)
Severity (Baseline severity score for children with ASD, a subgroup of the study population; Score range: 0–39)	15.67 (6.67) *N* = 30
Attended other NPAP group (Proportion) (e.g., group painting class)	0.19 (0.30)
Attended other PAP group (Proportion)	0.46 (0.37)
NPAP one-to-one (Proportion)	0.39 (0.38)
PAP one-to-one (Proportion)	0.55 (0.38)
Previous attendance in the program prior to baseline assessment (months)	26.13 (24.34)
Outcomes	Baseline Mean (SD)
Motor skills	29 (8.1)
Social adaptive skills	3 (2.4)
Leisure adaptive skills	4.9 (3.3)
Community participation	62.4 (19.2)
Home participation	79.8 (22)
Quality of life	91.8 (11.1)

a At least one of the cells had an expected count less than 5 and a fisher's exact test was used.

PAP, physical activity program; NPAP, non-physical activity program; DS, down syndrome; ASD, autism spectrum disorders; SD, standard deviation.

A high percentage of the participants presented with ASD: 31/42 (73.8%), with either single diagnosis (26/31) or in combination with another diagnosis (5/31). One study participant did not have a precise diagnosis, and another one did not report the diagnosis. The diagnoses were based on de-identified information provided by the parents in the demographic questionnaire, without medical verification. The mean age across the 42 participants was 107.1 (SD: 35.7) months at baseline; median age: 99.5 months (IQR: 55.8). Out of the 42 subjects, 34 (81%) were males and 8 were females.

### 3.1 Motor skills

Data were collected for 42 subjects at baseline, 39 (93%) with first 3-month follow-up and 24 (57%) with at least two follow-up assessments; only 13 (31%) subjects completed the third follow-up and 2 the fourth one. Due to the low number of attendances for these two last time-points, these observations are not reported in the descriptive analysis. However, these data were included in the LME models to have a maximum number of points for each regression.

The assessment of changes in motor skills scores (Table 2) shows that the change between baseline and follow-up 1 was statistically significant with both parametric and non-parametric tests. Other changes between first and second follow-up and from baseline to second follow-up were associated with large confidence intervals that indicate the absence of effects.

**Table 2:** Assessment of changes in motor skills scores using non-parametric and parametric tests.

Comparisons	Paired *T*-Test (parametric)	Wilcoxon signed rank test
Baseline vs. Follow-Up 1	Mean of paired differences: + 1.9 score point 95% CI: (0.32, 3.48) *p*-value: 0.020^a^ n: 39	*p*-value: 0.004^a^ n: 39
Baseline vs. Follow-Up 2	Mean of paired differences: −0.46 score points 95% CI: (−3.2, 2.3) *p*-value: 0.731 n: 24	*p*-value: 0.77 n: 24
Follow-Up 1 vs. Follow-Up 2	Mean of paired differences: −1.54 score points 95% CI: (−3.6, 0.27) *p*-value: 0.1 n: 24	*p*-value: 0.14 n: 24

a Indicating statistical significance.

The table shows the results of the Paired *T*-Test and Wilcoxon Signed Rank Test to examine changes between the assessments times.

For the whole study population, the simple LME model with duration of program exposure (month of exposure since baseline assessment) as the only independent variable, showed an average increase of 0.17 score-point (95% CI: −0.01, 0.34; *p*-value: 0.06) on motor skills score for every added month in the program. This average monthly change was 0.20 (95% CI: −0.03, 0.43; *p*-value: 0.08) in participants with ASD. In this subgroup, baseline severity scores seemed associated with motor skills since the slope of monthly change decreased by approximately 10% (from 0.20 to 0.18) after adjusting for this variable. However, the monthly average change in motor skills score was not modified by the severity score at baseline.

Attending other one-to-one or group PAP and NPAP did not affect the monthly change of motor function score. However, attending the other group-NPAP, after removing the outliers, showed an increase in the slope of monthly growth from 0.15 to 0.18 (+17%) when adjusting for another group-NPAP variable. Attending these other programs was also associated with a modification of the monthly average change of motor skills score. The scores decreased when a child attended another one-to-one PAP or NPAP; however, this score increased after removing the outliers.

In addition, after removing the outliers, for the whole study population, the average change in motor skill score for every additional month in the program decreased slightly from 0.17 to 0.15 (95% CI: −0.01, 0.31; *p*-value: 0.07) and that of the children with ASD decreased to 0.17 (95% CI: −0.06, 0.40; *p*-value: 0.14) from 0.20. In summary, the slope values were still positive, indicating an increase in the average motor skills scores as duration in the program increased. The father's education was also associated with the change in motor skills, responsible for an increase of 14% (from 0.15 to 0.17) of the monthly slope score, when added to the model. However, the monthly scores were very similar in each of the two categories of education (no effect modification).

### 3.2 Social adaptive skills

The data was collected from 38 subjects at baseline, 31 (82%) with first follow-up and 19 (50%) with at least two follow-up assessments; only 3 subjects (8%) completed the third follow up and no one completed the fourth. The change between the first and second follow-up scores was statistically significant.

For the whole study population, the simple LME model showed an average increase of 0.04 score point (95% CI: −0.04, 0.13; *p*-value: 0.31) on social skills score for every increasing number of months spent in the program and an average monthly increase of 0.13 (95% CI: −0.01, 0.26; *p*-value: 0.06) on social skills score in study participants with ASD.

We found some modification of the effect in different diagnoses categories. The estimated monthly change in social adaptive skills score was higher in children with ASD compared to children with other diagnosis. Also, attending another group-NPAP at one more follow up assessment increased the estimated monthly change in social adaptive skills outcome compared to not attending group–NPAP. No outliers were identified for the LME models**.**

### 3.3 Leisure adaptive skills

We had data for 37 subjects at baseline, 32 (86%) with first follow-up and 19 (51%) with at least two follow-up assessments; only 3 subjects (8%) made the third follow up and none made it to the fourth. For the whole study population, the simple LME model showed an average decrease of −0.04 score point (95% CI: −0.10, 0.02; *p*-value: 0.15) on leisure adaptive skills score for every increasing number of months spent in the program and the same monthly decrease of −0.04 (95% CI: −0.11, 0.03; *p*-value: 0.27) in study participants with ASD.

We did not identify any potential confounders when adjusted in the LME model. Possible interaction existed with attending other group PAP; leisure adaptive skills score increased when a child/youth attended the other program.

### 3.4 Community participation

We collected data from 41 subjects at baseline, 32 (78%) with first follow-up and 22 (54%) with at least two follow-up assessments; only 3 subjects (7%) made the third follow-up and only none made it to the fourth. For the whole study population, the simple LME model showed an average increase of 0.16 score point (95% CI: −0.30, 0.62, *p*-value: 0.48) on the community participation score for every increasing number of months spent in the program; there was also an average monthly increase of 0.21 (95% CI: −0.33, 0.75, *p*-value: 0.43) in study participants diagnosed with ASD.

After adjusting the LME model for the covariates of interest in the study gender, previous attendance, mother's education, NPAP one-to-one, PAP group and baseline severity were associated with the change in community participation. The monthly slope increased by 11% and 23% after adjusting for gender and previous attendance, respectively. Also, the monthly slope score in the simple LME model decreased by 18%, 34%, 14% and 66% after adjusting for the mother's education, NPAP one-to-one, PAP group and baseline severity, respectively. However, the estimated monthly change in community participation score was not modified by the covariates (i.e., no effect modification). In addition, age decreased the estimated monthly change in community participation score for every monthly increase in the age of a participant (i.e., effect modification).

### 3.5 Home participation

There was data available for 41 subjects at baseline, 33 (80%) at first follow-up and 21 (51%) with at least two follow-up assessments; only 3 subjects (7%) made the third follow up and no one made it to the fourth follow-up.

For the whole study population, the simple LME model showed an average increase of 0.16 score point (95% CI: −0.30, 0.63, *p*-value: 0.49) on the home participation score for every increasing number of months spent in the program and an average monthly increase of 0.25 (95% CI: −0.35, 0.85, *p*-value: 0.41) in the population with ASD. After adjusting the LME model for the covariates of interest, gender, age, mother's education, marital status, income, PAP-one-on-one, NPAP-one-on-one, PAP-group and NPAP-group were identified as being associated with the outcome and maybe potential confounders. After adjusting for gender, the monthly slope decreased by 10%. Adjusting for age, marital status, income, and mother's education, we found associations with the monthly slope of home Participation: a 21% decrease for age, a 10% decrease for marital status, a 62% increase for income, and a 11% increase for mother's education. We also observed a modification of the effects according to gender in favour of males.

Finally, we found that slope of month after adjusting for PAP-one-on-one, NPAP-one-on-one, PAP-group, and NPAP-group increased by 11%, decreased by 64%, decreased by 12% and increased by 10%, respectively.

### 3.6 Quality of life

The data was collected from 41 subjects at baseline, 33 (80%) with first follow-up and 22 (54%) with at least two follow up assessments; only 5 subjects (12%) made the third follow up and only 1 made it to the fourth follow-up. There was a statistically significant negative change between the baseline and the second follow -up assessment. For all study participants the simple LME model showed an average decrease of −0.22 score point (95% CI: −0.50, 0.06, *p*-value: 0.12) on QoL score for every increasing number of months spent in the program and an average monthly decrease of −0.10 (95% CI: −0.42, 0.22, *p*-value: 0.54) in the study participants with ASD.

After adjusting the LME model with the covariate of interest, father's education, diagnosis and baseline severity were identified as potential confounders. The slope for month in the simple LME decreased by 10% after adjusting for father's education, increased by 15% after adjusting for diagnosis and increased by 55% after adjusting for baseline severity.

Regarding possible effect modification, the estimated monthly change in the QoL score was higher in children whose father had high school/trade school training compared to the university education. For children with ASD, the estimated monthly change in the QoL score increased for every increase in baseline severity score. We also found a decrease in estimated monthly change in the QoL score with monthly increase in the age and monthly increase in the previous attendance in the ESMT™ program, respectively.

## 4 Discussion

Our findings show trends towards improved motor skills, as well as in home and community participation among children with NDID (mostly with ASD) in context of attending the ESMT™ program, over a 12-month follow-up. Although the observed possible gains could be the result of a child's normal development, we believe that the observed positive trend is mostly related to the specific precise scaffolding offered by the one-to-one personalized approach offered by the ESMT™ program. It is also possible that other contextual factors played an important role for some children (such as attending another programs), but it is not likely to influence most of the children in our study. A smaller trend of positive change was also observed in Social Adaptive skills. Overall, these findings are consistent with the ones observed in the retrospective study of the ESMT™ program published in 2019 (25). The discussion addresses results for each outcomes separately.

Motor skills showed a trend toward improvement (0.17 score point in BOT-2 SF for each month in the program) with even larger positive trend in children with ASD (0.20 score point). This improvement is supported by other studies. Najafabadi et al. 2018 (43) showed similar results when conducting a quasi-experimental study to investigate the effects of a 12-week SPARK exercise program (three 40-minute sessions per week), on the social and motor skills of 26 children with ASD between the ages of five and twelve. Similarly, Ketcheson et al. 2017 (44) conducted a non-RCT study of 20 children with ASD aged 4–6 years with a control group (*n* = 9) that did not receive the intervention. The intervention group participated in an 8-week intervention consisting of motor skill instruction for 4 h/day, 5 days/week. Statistically significant differences were found between groups on all three motor outcomes including locomotor, object control raw scores, and the total gross quotient (*p* ≤ 0.01). Finally, Sowa et al. 2012 (45) conducted a meta-analysis of 16 papers with a total of 133 participants with ASD, 4–14 years of age on the effects of physical exercise on three core symptom areas of ASD, motor, social, and communication skills. The results showed benefits of physical exercise on the participants’ motor and social functioning. Notably, individual-based interventions resulted in a greater improvement than group-based interventions.

Our findings with regard to the motor skills support a child-centered approach in which the training plans are consistently individualized to each child's unique needs, including their specific learning styles, weakness and strengths; this method works to maximize each child's potential for success through the ESMT™ program. This is also important to ensure the child remains motivated in the learning process. Children attending the ESMT™ program are assigned to a personal therapist and a set time for the school year to encourage consistent attendance and allow for relationship development with their therapist and other children/staff attending at the same time.

Social adaptive skills showed minimal change during the follow-up (0.04 score point). However, children with ASD progressed better (0.13 score point) than children with other diagnoses. This supports that the personalized approach in the ESMT™ program might have led to more direct communication and interaction between the child and the instructor resulting in the development of communication skills, and a better response to a child's special needs and improvements in a child's sense of trust, especially important for children with ASD. This result is particularly interesting because the ESMT™ program does not offer a structured interactional strategy to promote social development among children. Yet, their personalized approach led to the progress in social adaptive skills, especially in children with ASD. It is also important to note that social adaptive skills maybe affected by other factors such as socioeconomic status or parental education.

Other studies have shown positive impacts of personalized adapted programs to improve social adaptive skills among children with ASD. Zanobini and Solari 2019 (46) found positive results in social outcomes from a one-to-one swimming program using a quasi-experimental design including pre-test, post-test and after 6 months follow up. They recruited 25 children with ASD, 3–8 years old. The analysis revealed a significant Time x Group interaction for social outcome where the experimental group showed higher improvement compared to the control group. In contrast, Bremer et al. 2014 (47) studied the effectiveness of a 12-week fundamental motor skill intervention for 9 children aged 4 years old with ASD using a 1:1 or 1:2 ratio and RCT design. There were statistically significant changes between the control and the experimental groups in motor improvements but not in adaptive behavior or social skills.

Community participation indicated possible gains over time (0.16 score point). This positive change was larger in children with ASD (0.21 score point). Similar positive trends have been shown in several studies. For instance, Ajzenman et al. 2013 (48) in a pre-post pilot study aimed at determining the effect of 12 weeks hippotherapy (HPOT) on motor control, adaptive behaviors, and participation among children with ASD. They included a small sample size of 7 children aged 6–12 years, with no control group. Based on these interviews they found significant increases in participation in daily activities, self-care, low-demand leisure, and social interaction. However, Zwicker et al. 2015 (49) conducted another pre-test/post-test study to investigate the effects of a summer camp intervention for 11 children with Developmental Coordination Disorder (DCD) who attended a 2-week camp twice, one year after the other, with 2:1 child to staff ratio. They found no significant changes in community participation. Additionally, Cleary et al. 2017 (50) conducted a two-group single-blinded phase I randomized controlled trial on a 9-week aerobic exercise program for 19 young people aged 8–18 with cerebral palsy using a 1:1 or 1:2 staffing ratio. Results showed no difference between groups in community participation and preference. The possible positive change in community participation after attending the ESMT™ program may be due to its participant-centered, multidimensional approach and the sense of trust that progressively develop over time between the child and their personal coach. This approach promotes more effective communication with the instructors, builds self confidence, and supports the ability to speak and share opinions. Together, these results may contribute to increased community participation.

However, the change in this outcome could also be connected to other factors such as family interest in participating in community activities, family culture, income, etc. We also observed a positive trend in home participation overtime similar to community participation (0.16 score point). This positive change was higher in children with ASD (0.25 score point). This positive trend is likely supported by the same factors that support community participation to enhance socialization.

A negative trend for QoL was observed during the course of the study (−0.22 and −0.10 score points) for all participants and children with ASD, respectively. This negative trend was not expected, but it has been observed in other studies. In a randomized controlled trial of a 9-week aerobic exercise program in a young population of children with cerebral palsy (*n* = 19), Cleary et al. 2017 (50) failed to show a significant difference in the QoL outcome measure within the intervention group. Oriel et al. 2012 (51), studied the benefits of an inclusive community-based aquatics program which ran for 8 weeks with 1:2 adult to child ratio using a quasi-experimental design. They included *N* = 23 children with and without disabilities. There was a large age discrepancy between the two groups: disability group mean 12.3 years, without disability group mean 6.77 years. Measures included QoL, self-concept, and peer acceptance. They observed a significant decrease in happiness in children with disabilities. Children with NDID also demonstrated a significant decrease in the School subsection of the PedsQL 4.0 compared to neurotypical children. Some studies, however, did show a positive change in QoL. As an example, Ansa et al. 2021 (52) studied the effect of an 8-week community-based functional aerobic training on the motor performance and QoL for 10 children with Cerebral Palsy 12–16 years of age. Unlike the other studies, they found significant improvement in QoL. Changes in QoL score may be difficult to interpret because it is affected by multiple factors around the child that were not considered in this study. For instance, a model proposed to understand the factors that influence the QoL in ambulatory children with Cerebral Palsy, adapted from the framework of the International Classification of Functioning (ICF), emphasizes the importance of activity capacity, activity performance, body structure and function, health conditions, participation, and personal factors such as age or satisfaction (53). For example, the child's motor function could improve while the QoL regresses because of a problem with the school experience, or a personal. negative experience. Factors related to the family such as parental illness, conflict with siblings or divorce could also impact the child's QoL negatively as per family transactional interaction (54). In addition, the change in QoL is not expected to follow at the same pace as the changes in motor skills; observation for periods longer than one year may be required to captured possible delayed improvement. Another factor to consider is the difficulty for some parents to complete the KIDSCREEN-27 to describe their child's quality of life. For instance, parents' scoring may be affected by some disappointment when they consider their child to have made little progress. Finally, the KIDSCREEN-27 and similar tools were developed mainly for neurotypical children and may not be sensitive enough to capture changes in children with NDIDs. It was not fully validated to reflect what represents quality of life for children with NDIDs.

Our analysis also investigated the impact of children's and families' baseline characteristics, as well as the child's participation in other physical or not-physical activities on observed changes in measured outcomes. However, all findings regarding the possible confounders and effect modifiers are to be considered with caution because of the small sample size and selected population. Table 3 shows the list of potential confounder and effect modifiers identified for each outcome. Attending other one-to-one PAP has been shown to decrease the monthly motor skills score. This effect seemed to be opposite for attending other group PAP. Those differential effects might reflect the fact that children with more severe condition may attend more one-to-one programs than group programs and at the same time, these children have more difficulty to improve their skills, despite attending more programs. Regarding the positive effect of attending other group PAP, there maybe two possible explanations: one is that attending more group PAPs may stimulate more participation, hence more engagement in the PAP and more progress. The second reason maybe that families of children who were attending more activities might be generally more involved in their child's progress, which might result in an improvement in their outcome scores. These hypotheses should be verified in future studies with larger sample sizes and mixed method to better capture the process of improvement and related factors. Observing the positive impact of a younger age for community participation and QoL outcomes, if verified, may highlight the importance of early interventions. Finally, the positive impact of shorter previous attendance on QoL maybe the result of the child not reaching the plateau effect yet. Gymnastics is recognized as a physical literacy program which helps children to develop fundamental movement skills such as jumping, climbing, running, balancing and coordination. In early stages of physical development, the emphasis is on physical literacy improvements where there is generally faster development than in later stages where the emphasis is on skill development and the refinement of more difficult skills. This can lead to a plateau effect which is associated with a period of stagnation or leveling off in progress and is a natural phenomenon in various aspects of life, including learning, exercise and personal growth.

**Table 3:** List of potential confounders and effect modifiers for each outcome.

Outcome	Confounder	Effect modifier Positive impact (+) Negative impact (-)
Motor skills	Baseline severity (for ASD) Attending other group NPAP Father education	Attending other group PAP (+) Attending other one-to-one PAP (-) Attending other one-to-one NPAP (-)
Social adaptive skills	N/A	ASD (+) Attending other group NPAP (+)
Leisure adaptive skills	N/A	Attending other group PAP (+)
Community participation	Gender Previous attendance in the ESMT™ program Mother's education Attending other one-to-one NPAP Attending other group PAP Baseline severity (for ASD)	Age (-)
Home participation	Gender Age Marital status Attending other one-to-one PAP Attending other one-to-one NPAP Attending other group PAP Attending other group NPAP Mother's education, Income	Male (+)
Quality of life	Father education Diagnosis Baseline severity (for ASD)	Age (-) Previous attendance in the ESMT™ program (-) Male (+)

N/A, not applicable: No potential confounder identified.

In summary, our investigation suggests possible benefits of the ESMT™ program for physical abilities and participation in both community and home settings, though these results should be interpreted with caution in context of an exploratory study. The positive changes may reflect the program's inclusive atmosphere, where children of all abilities learn similar skills at their pace. The activities are carefully scaffolded to each participant's developmental level, ensuring that all participants are in their zone of proximal development, whether that be learning to walk or mastering a double back salto. This extremely large range in learning has one thing in common: all children are being reinforced by their effort rather than their skill level. Children of all abilities share laughter, tears, and cheers of success, while cheering on those that are struggling. That is what allows all the participants, regardless of ability and differences among them, to feel a sense of camaraderie in the gym. The common thread is that all participants are learning at their own unique pace and based on their specific needs, as well as the personal and family related factors. This process is in line with the sociocultural learning theory of child development that emphasizes the paramount importance of the social context, community involvement and the use of a large range of artefacts and language as tools for optimal learning (55). The learning process is continuous due to the stimulation of increasingly more complex psychological functions such as, e.g., curiosity, logical thinking, problem solving and complex cognition. Success at completing tasks is a source of self-esteem and self-confidence; this promotes further motivation to acquire new knowledge along with physical and social skills.

The findings of this study should be considered in light of several limitations. First, bias from loss to follow-up may have influenced the results, as many participants dropped out for reasons like personal health and family issues, surgery, or study demands (e.g., completing multiple questionnaires). We compared baseline and follow-up 1 scores, as well as the percentage change between the two assessments, between the dropout group (those who left after the first follow-up) (Supplementary Table S1) and the non-dropout group (those who remained) (Supplementary Table S2). This showed that the dropout group made greater progress while they were in the study, meaning our analysis focused on children with slower progress, likely underestimating improvements. However, for community and home participation outcomes, the results might be biased toward being overly optimistic since the dropout group showed less progress.

A second limitation is the absence of a control group not receiving the personalized intervention. It is then difficult to assess the specific effects of attending the program and interpret the meaning of an improvement of 0.17 or 0.20 score point in the BOT-2 scale. Selecting a control group identical to the study participants and not receiving any interventions is almost impossible; from our experience, most children with NDID receive some kind of interventions in different settings, special schools, etc. making the comparison difficult and not valid. Further, comparison with the progression of children with neurotypical development is irrelevant. Finally for the same reason of huge heterogeneity among children attending ESMT™, it is not possible to determine a minimal clinically important difference (MCID) that could be used as valid standard comparator. Consequently, we used the change in score as the value that helped determining the progress, and we observed that children with ASD improved faster than the group that had other diagnosis.

The small sample size is another limitation that limited the ability to detect statistical significance and restricted the development of multivariate models to only include covariates one at the time to assess their impact on the main association. The lack of blinding, due to the study's nature, could also be a limitation, although data collection was conducted rigorously by a single assistant within the child's usual program. Another limitation is the limited description of the concomitant interventions that children may receive when they attend other community programs. We only collected the information regarding attendance of such programs but no information regarding the specific features of the programs. We also did not collect any information regarding the possible practice of physical activity at home. Practicing at home or attending other programs may have a positive effect regarding child learning and development; however, attending several programs may also reflect the family anxiety regarding their child's development. One limitation is a possible “parental report bias”, as questionnaires were completed by parents instead of the children, due to the children's limited capacity to respond as a result of their NDID. Lastly, while most findings were positive, previous research indicates optimal physical activity outcomes require participation three times a week (56), while most children in the ESMT™ program participated 1–2 times a week. The cost of one-to-one programming, not covered by Canada's medical system, also limits the feasibility of more frequent sessions for many families.

Despite these limitations, our findings show in a reliable way the positive impact of the one-to-one PAP on motor skills development and social participation at home and in the community. Further investigations are still needed to include a larger sample size and collect and monitor the child's and family's personal factors in more detail. A comparison with a control group should also be included to account for the effects of normal development.

Finally, it may be possible to enhance the exposure to the inclusive and motivating environment of the ESMT™ intervention by including other professionals to the team such as a Speech and Language Pathologist, Behavior Consultant and Physiotherapist, which may lead to even greater results. It would be the beginning of a Hub model for the treatment of the neurodiverse population with all professionals working as a team. The effects of this intervention model on children and adolescents with ASD should be considered in future studies.

One final point of attending the ESMT™ intervention is the high cost that may limit access to the program. Although access to the facility and use of various equipment are free of charge, as they are covered by the general gym program, the sessions' fees reflect the program's expenses for management and paying professionals for individualized one-to-one coaching. We hope this article promotes interest for further development of one-to-one programs and help advocating for public financial support for such programs.

## 5 Conclusion and future directions

This exploratory cohort study was conducted with children and youth with NDID (mostly ASD) while attending the ESMT™ program to assess changes in motor and social adaptive skills and changes in a wide range of other outcomes. Despite the major limitations, this prospective cohort study suggests that the training program may support improvements in motor skills, community and home participation. These findings also suggest that the benefits of attending this community-based PAP may go beyond the motor skills development. Children with ASD may benefit the most from this personalized program, especially among those with higher levels of severity. ASD is a well-documented disease that can be generally characterized by limited social communication and “restricted, repetitive sensory-motor behaviours”, difficulty in relationship building, and over- or under-reaction to various environmental stimuli (1, 57). For children with ASD, the one-to-one approach maybe the preferred intervention as it provides opportunity for more effective scaffolded communication with the instructors in an environment that can be better adapted to their needs (e.g., less stimulation by sound or light). Finally, the observed changes were associated and affected by several other child and family related factors. Health care providers may consider those factors when suggesting participation in community-based PAPs for children with NDID. They may also use the results of the present study to organize the care plan and data collection for children and youth with NDID. Families are also encouraged to have their children and youth with NDID participate in community-based PAPs. Finally, our study suggests that a multisystem approach embedded in social contexts could be included within the standard-of-care treatment for ASD (22).

## Data Availability

The raw data supporting the conclusions of this article will be made available by the authors, without undue reservation.
