# Supplementary material for: Attending a one-to-one child-centered movement therapy program improves multiple outcomes among children with neurodevelopmental disabilities: an exploratory prospective cohort study
Source: Front Pediatr. 2025 Nov 13;13:1623686. doi: 10.3389/fped.2025.1623686 (PMC12658744; doi:10.3389/fped.2025.1623686)
Supplement: Supplementary file 1 [file Table1.docx]

Supplementary Material

**Table S1.** Summary statistics for dropped out group for motor skills outcome

|  | **BL** | **FU1** | **BL-FU1** |
| --- | --- | --- | --- |
| Sample size (count) | 15 | 15 | 15 |
| Missing (count) | 0 | 0 | 3 |
| Mean | 26.6 | 29.8 | +13.1% |
| SD | 4.7 | 5.2 | +16.3% |
| Median | 27 | 31 | +12% |
| IQR | 6.5 | 6.5 | +25% |

Abbreviations: BL – Baseline; FU1 – First follow up visit; FU2 – Second follow up visit; IQR – Interquartile range; SD – Standard deviation.
