# Supplementary material for: Attending a one-to-one child-centered movement therapy program improves multiple outcomes among children with neurodevelopmental disabilities: an exploratory prospective cohort study
Source: Front Pediatr. 2025 Nov 13;13:1623686. doi: 10.3389/fped.2025.1623686 (PMC12658744; doi:10.3389/fped.2025.1623686)
Supplement: Supplementary file 2 [file Table2.docx]

Supplementary Material

**Table S2.** Summary statistics for non-dropped out group for motor skills outcome

|  | **BL** | **FU1** | **FU2** | **BL-FU1** | **BL-FU2** | **FU1-FU2** |
| --- | --- | --- | --- | --- | --- | --- |
| Sample size (count) | 24 | 24 | 24 | 24 | 24 | 24 |
| Missing (count) | 0 | 0 | 0 | 0 | 0 | 0 |
| Mean | 30.6 | 31.7 | 30.1 | +5.2% | +1.6% | -3.1% |
| SD of Mean | 9.7 | 8.5 | 6.9 | +13.2% | +16.4% | +13.2% |
| Median | 27 | 31.5 | 29.5 | +6.1% | +2% | -2.8% |
| IQR | 9.3 | 11.5 | 8.00 | +14.1% | +21.4% | +15% |

Abbreviations: BL – Baseline; FU1 – First follow up visit; FU2 – Second follow up visit; IQR – Interquartile range; SD – Standard deviation.
