# Supplementary material for: Attending a one-to-one child-centered movement therapy program improves multiple outcomes among children with neurodevelopmental disabilities: an exploratory prospective cohort study
Source: Front Pediatr. 2025 Nov 13;13:1623686. doi: 10.3389/fped.2025.1623686 (PMC12658744; doi:10.3389/fped.2025.1623686)
Supplement: Supplementary file 3 [file Datasheet1.docx]

Supplementary Material

Description of the ESMT^TM^ Process

The ESMT^TM^ program occurs in a 16,000 square foot gymnastics facility which offers a wide range of programming from beginners (18 months) to high performance women’s artistic and trampoline programs. As such the equipment is state of the art. For example, there are 5 trampolines (four above ground and one in-ground all of which are designed for high performance training.

The categories we use in the scale are all associated with gymnastics equipment such as:

- 40x40 sprung floor and a rod floor
- 5 trampolines
- 8 balance beams some 6” wide and some 4” wide a variety of heights floor to 125 cm in height. The 4 inch beams can all be lowered and raised in height by a crank.
- Parallel Bars
- Single Bars
- Trapeze
- Air track: This is a station specifically designed for ESMT^TM^ and preschool classes which includes a slide and a bouncy 30 jumping surface
- Climbing apparatus: Dowel ladder, wall bar, knotted rope, stairs
- Rolling and rocking equipment: Dome, incline mats, Pac Man, rollers of various sizes
- Manipulatives: Racquets, balls, scarves, balloons, beanbags
- Blocks both 25 cm and 50 cm high

In addition to the categories, we have reward activities which can be earned at the end of the session such as a large foam pit, hammock and special education swings which can be used by all ages and sizes.

Table below shows the whole process of building motor skills with different gymnastic equipment.

| **Category name (used on assessment checklists)** | **Category description** | **Examples of skills in a sample of stages (St.)** |
| --- | --- | --- |
| Floor | 40x40 sprung floor and a rod floor, carpeted floor surfaces | St. 6: Hops forward L&R 5x  St. 7: Long jump 1.2 body height  St. 7: 10x skipping on the floor with contralateral arm action |
| Trampoline | 5 trampolines | St. 4: Jumping 20x consecutively  St. 5: Seat drop to stand in balance  St. 6: sequence: seat drop, doggy drop to seat drop to stand in balance |
| Beam | 8 balance beams some 6” wide and some 4” wide a variety of heights floor to 125 cm in height. The 4 inch beams can all be lowered and raised in height by a crank. | St. 4: on a 6” beam 40cm off the ground walks the length of a regulation beam independently  St. 5: walks sideways L&R on a 6” beam raised hip height  St. 6: Walks forward on a 4” beam 20cm off the floor |
| Parallel Bars | Parallel Bars | St. 5: Bear walk Frwd/Bkwd  /Side L&R  St. 6: Crab Walk Frwd/Bkwd  St. 7: Tuck hold, knees at hip height for 10 sec. |
| Single Bar | Single Bar | St. 6: Holding onto a hip height bar walk feet up the wall to an inverted position and hold for 5 sec.  St. 8: Spin the cat independently to land on feet  St. 9: Jump to a front support hold 3 sec and execute a forward roll dismount. |
| Trapeze | Trapeze | Stage 4: swings from and elevated box out and back independently  Stage 5: Climbs to a stand on a box and swings independently on the trapeze to land on 2 feet with stability  Stage 10: Swing out and back with legs in a straddle position with legs straight and at hip height |
| Airtrack | Air track: This is a station specifically designed for ESMT^TM^ and preschool classes which includes a slide and a bouncy 30 jumping surface | St. 4: Jumps over 3 ropes on the airtrack without touching the ropes  St: 5: Climbs the rope on the airtrack  St: 6: Jump series: 3 jumps over rope to immediate dive roll onto an incline mat |
| Wallbar (example) | Falls into a broader topic of climbing apparatus, including Dowel ladder, wall bar, knotted rope, stairs | St. 4: Climbs up the wall baron the airtrack to a seated position on the top  St. 5: Climbs up the wall bar 5 rungs and down 5 rungs  St. 6: Climbs the wall bar up and down using contralateral hands and feet 5 rungs each way |
| Rolling | Falls into a broader topic of rolling/rocking apparatus, including Dome, incline mats, Pac Man, rollers of various sizes | St. 4: From a stand on an incline reaches down and executes a forward roll  St. 5: Log roll L&R down the incline arms straight and overhead and legs together and straight  St. 6: Executes a backward roll down the incline mat with proper hand placement to land on feet |
| Ball Handling | Manipulatives: Racquets, balls, scarves, balloons, beanbags  (some of these are also included in the “Floor” and “Beam” categories) | St. 6: Bats a balloon 5x consecutively with both L&R hands on the floor  St. 7: Walks forward on a 6” beam while throwing and catching a beanbag  St. 8: On a 6” beam jumps over a series of bean bags the length of the beam with no pauses or stops raised 1m |
| Box | Blocks both 25 cm and 50 cm high | St. 4: 3x jump off a 25 cm block from 2 feet to land on 2 feet with a stable (motorcycle) landing  St. 7: Execute a plyometric sequence on 3 25 cm blocks and an incline mat: up-down- up-down-up-down-dive roll with no stops or pauses |
