# Supplementary material for: Attending a one-to-one child-centered movement therapy program improves multiple outcomes among children with neurodevelopmental disabilities: an exploratory prospective cohort study
Source: Front Pediatr. 2025 Nov 13;13:1623686. doi: 10.3389/fped.2025.1623686 (PMC12658744; doi:10.3389/fped.2025.1623686)
Supplement: Supplementary file 4 [file Datasheet2.docx]

Supplementary Material

Case Study

Initial Assessment 8-year-old male

Description provided by the parent:

- - Speech & Language Disorder: Limited Verbal Speech
  - Global Developmental Delay
  - Motor- Poor balance and coordination
    - Walks independently
    - Low muscle tone

Notes from the initial assessment (see Supp. “ESMT^TM^ program description”):

- It was observed that the child could not jump, when asked they used a gallop step
- Fear of heights, the child would not climb even one step of a dowel ladder
- Fear of inversions, the child did not like to reach down to the floor

The following chart represents some of the Initial Assessments Report and the first Biannual Assessment Scores (approximately 6 months later) for the same skills.

| **Skill & Description** | **Assessment Criteria** | **Scores** | | **Initial Assessment Score** | **1st Biannual Assessment Score (approx. 6 months later)** |
| --- | --- | --- | --- | --- | --- |
| Category 20: From a stand on the incline, reaches down and executes a forwards roll | Hands reach down to the floor | 0.25 | | 0.25 | 1 |
|  | Chin tucked | 0.25 | |  |  |
|  | Initiates rolling motion | 0.25 | |  |  |
|  | Maintains rotational axis | 0.25 | |  |  |
| Category 6: Walks forwards up and down an inclined "6 inch beam" for 2.4m each way | Able to perform with assistance | U 0.125 | D 0.125 | up 0.25 | 1 |
|  | 1/3 length independently | U 0.125 | D 0.125 |  |  |
|  | 2/3 length independently | U 0.125 | D 0.125 | down 0.25 |  |
|  | Entire length independently | U 0.125 | D 0.125 |  |  |
| Category 7: Walks the length of a regulation beam (4.8m) on a "6 inch beam" elevated a minimum of 40 cm | Able to perform with assistance | 0.25 | | 0.5 | 1 |
|  | 1/3 length independently | 0.25 | |  |  |
|  | 2/3 length independently | 0.25 | |  |  |
|  | Entire length independently | 0.25 | |  |  |
| Category 11: Jumps on the trampoline from 2 feet to 2 feet for more than 20 consecutive bounces | 9 jumps | 0.25 | | 0 | 1 |
|  | 14 jumps | 0.25 | |  |  |
|  | 19 jumps | 0.25 | |  |  |
|  | 20 jumps | 0.25 | |  |  |
| Category 9: Jumps from 2 feet over a rope on the AirTrack 3 times | Able to perform with assistance | 0.25 | | 0 | 1 |
|  | 1st time independently | 0.25 | |  |  |
|  | 2nd time independently | 0.25 | |  |  |
|  | 3rd time independently | 0.25 | |  |  |
| Category 16: Climbs the dowel ladder on the AirTrack to a seated position on top | Independent hands | 0.25 | | 0 | 1 |
|  | Independent feet | 0.25 | |  |  |
|  | Climbs full distance | 0.25 | |  |  |
|  | Maneuvers to seated position at the top | 0.25 | |  |  |
